# Potential roles of melatonin and ABA on apple dwarfing in semi-arid area of Xinjiang China

**DOI:** 10.7717/peerj.13008

**Published:** 2022-03-31

**Authors:** Tianci Yan, Chuang Mei, Handong Song, Dongqian Shan, Yanzhao Sun, Zehui Hu, Lin Wang, Tong Zhang, Jixun Wang, Jin Kong

**Affiliations:** 1 College of Horticulture, China Agricultural University, Beijing, China; 2 Sanya Institute of China Agricultural University, Sanya, Hainan, China; 3 Scientific Observing and Experimental Station of Pomology (Xinjiang), Ministry of Agriculture, Urumqi, Xinjiang Uygur Autonomous Region, China; 4 Institute of Horticultural Crops, Xinjiang Academy of Agricultural Sciences, Urumqi, Xinjiang Uygur Autonomous Region, China

**Keywords:** Apple, Dwarfing, Melatonin (MT), Abscisic acid (ABA), Drought stress

## Abstract

Dwarfing is a typic breeding trait for mechanical strengthening and relatively high yield in modern apple orchards. Clarification of the mechanisms associated with dwarfing is important for use of molecular technology to breed apple. Herein, we identified four dwarfing apple germplasms in semi-arid area of Xinjiang, China. The internodal distance of these four germplasms were significantly shorter than non-dwarfing control. Their high melatonin (MT) contents are negatively associated with their malondialdehyde (MDA) levels and oxidative damage. In addition, among the detected hormones including auxin (IAA), gibberellin (GA), brassinolide (BR), zeatin-riboside (ZR), and abscisic acid (ABA), only ABA and ZR levels were in good correlation with the dwarfing phenotype. The qPCR results showed that the expression of melatonin synthetic enzyme genes *MdASMT1* and *MdSNAT5*, ABA synthetic enzyme gene *MdAAO3* and degradative gene *MdCYP707A*, ZR synthetic enzyme gene *MdIPT5* all correlated well with the enhanced levels of MT, ABA and the reduced level of of ZR in the dwarfing germplasms. Furthermore, the significantly higher expression of ABA marker genes (*MdRD22* and *MdRD29*) and the lower expression of ZR marker genes (*MdRR1* and *MdRR2*) in all the four dwarf germplasms were consistent with the ABA and ZR levels. Considering the yearly long-term drought occurring in Xinjiang, China, it seems that dwarfing with high contents of MT and ABA may be a good strategy for these germplasms to survive against drought stress. This trait of dwarfing may also benefit apple production and breeding in this semi-arid area.

## Introduction

Apple (*Malus domestica*) is one of the top four fruits with the largest planting areas globally (Cohen et al., 2007). For apples, dwarfing is the major breeding objective to shorten juvenile period, increase yield and maintain mechanical strengthening for the high yield (Bulley et al., 2005; Smolka et al., 2010). Although years of crossing and selection to breed apple cultivar for ideal tree architecture, the elite of spur-type apple cultivar combined with other good horticultural traits is still a goal for apple breeders (Zheng et al., 2018). Many efforts have been made to uncover dwarfing mechanism, however, the complex underlying interactions between plants and environments remain unclear (Cohen et al., 2007).

The regulatory effects of hormones, cytokines (CKs), auxin (IAA), brassinosteroids (BRs), and gibberellins (GAs) on dwarfing have been extensively studied (Waldie & Leyser, 2018; Chen et al., 2019). Many genes involved in GA, IAA and BR synthesis, degradation, transportation and signaling have been identified (Davière et al., 2014; Ma et al., 2016). In apple, these hormones also play critical roles in dwarfing (Tong et al., 2014; Unterholzner et al., 2015; Guo et al., 2020). However, different from herbal plants, which flower earlier under severe stresses to end the life cycle, apple trees have developed more complex dwarfing mechanisms (Hatayama & Takeno, 2003; Xu et al., 2014; Minh-Thu et al., 2018) to better suit the long lasting drought stresses. Apple trees usually retard their normal-growth to enhance defense, which leads to a stress-defensive dwarfing phenotype (Liu et al., 2021).

Drought stress is increasing with the global warming, which severely affects apple yield. To adapt the upcoming global warming, it is important to breed dwarfing apple to balance the growth and drought tolerance to maintain its yield. In recent years, melatonin has been frequently reported to exhibit important roles involved in both drought tolerance and plant development (Zhang et al., 2015; Sun et al., 2021; Ahmad et al., 2021). The mechanism may attributed to its antioxidative activity. For example, long-term drought stress in the main apple production area of China, unavoidably results in secondary oxidative damage indicated by increased Reactive Oxygen Species (ROS) and malondialdehyde (MDA) level (Zuo et al., 2014; Huang et al., 2020). The ROS burst induces the biosynthesis of melatonin (MT), a well-known strong ROS scavenger in response to drought stress (Zuo et al., 2014; Wang et al., 2017). When apple trees are long-term-stressed, the induced MT might mediate a stress-defensive dwarfing. However, more evidence needs to be explored.

Melatonin is an amphiphilic molecule (Tan et al., 2012), which can freely cross cell membranes to any organelles including the chloroplast, nucleus, and mitochondria. MT metabolites can also synergistically scavenge ROS, resulting in amplified antioxidant capacity (Sun et al., 2021; Siddiqui et al., 2019). The biosynthesis pathway of melatonin in plants has been well-documented. An amino acid tryptophan is the precursor of melatonin synthesis. The tryptophan is catalyzed successively by tryptophan decarboxylase (TDC), tryptopine-5-hydroxylase (T5H), serotonin-N-acetyltransferase (SNAT) and N-acetylserotonin methoxy transferase (ASMT) to form melatonin, among which SNAT and ASMT are the rate-limiting enzymes in plant (Tan et al., 2012; Zhang et al., 2019; Sun et al., 2021).

ABA, a so-called stress hormone, functions in promoting stoma closure to reduce water lost through transpiration regulation (Shu et al., 2016; Long et al., 2019; Dou et al., 2021). Therefore, ABA inhibits plant growth *via* down-regulating photosynthesis (Jiang et al., 2021). ABA level is induced by drought stress. Its metabolic pathway has been well studied (Shu et al., 2016; Ren et al., 2018; Pang et al., 2019). Shi et al. (2021) reported that the drought-induced OsAAO3 regulates rice growth and development. The dehydration responsive *CYP707A*, encoding a key enzyme in ABA catabolism limits the plant growth for the purpose to survive under drought stress *via* reducing the expression of genes such as *RD22* and *RD29* (Matilla, Carrillo-Barral & Rodríguez-Gacio, 2015; Yu et al., 2020).

Cytokinin which is also in response to drought resistance *via* triggering *RR1* and *RR2*-involved signaling (Nishiyama et al., 2011), is reported to negatively regulate apple dwarfing *via* regulating cell division. The *Arabidopsis* mutants of *IPT5b* gene, encoding for a ZR synthetase, exhibit reduced level of the endogenous active forms and severe dwarfism (Tokunaga et al., 2012). In apple, the low expression of *IPT5b* leds to poor zeatin biosynthesis and dwarfing (Feng et al., 2017). The over-expression ZR degradation gene of *CKX1* in transgenic Arabidopsis and tobaccos decreased ZR level, enhanced stress tolerance and retarded shoot growth (Werner et al., 2010; Macková et al., 2013).

Herein, we have collected four apple dwarfing germplasms with shortened nodular length in the semi-arid area of Xinjiang, China. We have not found a good correlation between these dwarfing germplasms with the well-known hormones involved in dwarfing, such as GA, BR and auxin. However, the contents of stress induced hormone ABA and melatonin exhibit positive correlation with these four dwarfing apple germplasms. In addition, ZR content is also decreased in all the germplasms and this suggests its involvement in dwarfing. Considering a typical long-term drought season in this area, apples grown in this area will experience high level oxidative tissue damage and they will increase the levels of stress-responsive molecules, MT and ABA by upregulating the expression of their synthetic and signaling genes, it infers a stress-defensive dwarfing mechanism mediated by melatonin and ABA, as an important strategy to survive drought stress in the semi-arid area of Xinjiang.

## Materials and Methods

### Plant materials

The leaves and shoots were collected from the annual branches of the four dwarfing apple germplasms (Dwarf1-4) and a non-dwarfing germplasm (GB2), all the germplasms grafted onto 8-year-old Xinjiang Wild Apple (*Malus sieversii* (Lebed.) Roem.) in northern Xinjiang, China (82°51′46″E and 43°15′49″N, altitude 1,365 m) which belongs to semi-arid area, with an annual precipitation of proximately 200 mm (Chen et al., 2015). The germplasms in this semi-arid area are under long-term drought stress. The internodal distance of four spur-type apple germplasms were measured, with the non-dwarfing germplasm ‘GB2’ used as control, and the second node of the annual branch was the basal node for node counting. The leaf-samples were quickly frozen with liquid nitrogen, and then were kept at −80 °C till to use. The cell number of stem cortical parenchyma cells were observed using paraffin sections made by Servicebio Co. (Wuhan, Hubei, China) and scanned by CaseViewer2.3. Three different regions (250 × 75 μm) of the middle of internodes were selected for observation.

### Detection of malondialdehyde (MDA)

A total of 1 g leaves from the five apple germplasms (GB2, Dwarf1-4) were homogenized for MDA detection, respectively according to Zhao et al. (2013). Each experiment was independently repeated three times.

### Extraction and analysis of phytohormones of abscisic acid (ABA), indole-3-acetic acid (IAA), Gibberellin A3 (GA3), trans-zeatin-riboside (ZR) and brassinosteroids (BR)

A total of 0.5 g leaves from the five apple germplasms (GB2, Dwarf1-4) were used for extraction of ABA, IAA, GA3 and ZR, as described by Yang et al. (2001), and BR as described by Xin et al. (2013), respectively. ELISA analysis of ABA, IAA, GA3, ZR and BR were carried out according to Yang et al. (2001). Each experiment was independently repeated three times.

### Extraction and analysis of melatonin

A total of 0.4 g leaves from the five apple germplasms (GB2, Dwarf1-4) were ground to a fine powder with liquid nitrogen for melatonin extraction and analysis by HPLC as described by Zhao et al. (2013), respectively Each experiment was independently repeated three times.

### RNA extraction and qRT-PCR analysis

To detect the expression of melatonin synthetic enzyme genes (*MdASMT1* and *MdSNAT5*), ABA synthetic enzyme gene (*MdAAO3*), degradative enzyme gene (*MdCYP707A*) and ABA signaling genes (*MdRD22* and *MdRD29*), ZR synthetic enzyme gene (*MdIPT5*) and ZR signaling genes (*MdRR1* and *MdRR2*) by qPCR analysis, total RNA was isolated from 0.1 g leaves of the five apple germplasms (GB2, Dwarf1-4), respectively with EASY spin Plant RNA Kit (Aidlab Biotechnologies, Beijing, China). cDNA was obtained by reverse transcription, using TUREscript RT MasterMix (Aidlab Biotechnologies, Beijing, China) following the protocol, as template for qRT-PCR. Each experiment was independently repeated three times.

The specific primers of *MdAAO3*, *MdCYP707A*, *MdIPT5, MdRR1, MdRR2, MdRD22* and *MdRD29* genes were designed by Primer 5 software according to the coding sequences from GDR (https://www.rosaceae.org/species/malus/all), and the specific primers of *MdASMT1* and *MdSNAT5* genes were used as described by Zuo et al. (2014) and Wang et al. (2017), respectively. The Actin gene was used as the internal control. All the primers are shown in Table S2. qRT-PCR was performed with TB Green® Premix Ex Taq (Takara, Dalian, China) on an ABI 7500 real-time PCR machine (ABI, Carlsbad, CA, USA) according to Zheng et al. (2018). Each experiment was independently repeated three times.

### Statistical analysis

The data were expressed as mean ± SEM and analyzed by one-way analysis of variance (ANOVA) followed by Fisher’s Least Significant Difference (LSD) test. *p* < 0.05 was considered statistically significant. The SPSS 25.0 software (IBM, Armonk, NY, USA) was used for statistical computations.

## Results

### The variation of nodular distance between non-dwarfing and dwarfing apple germplasms

**Figure 1:**
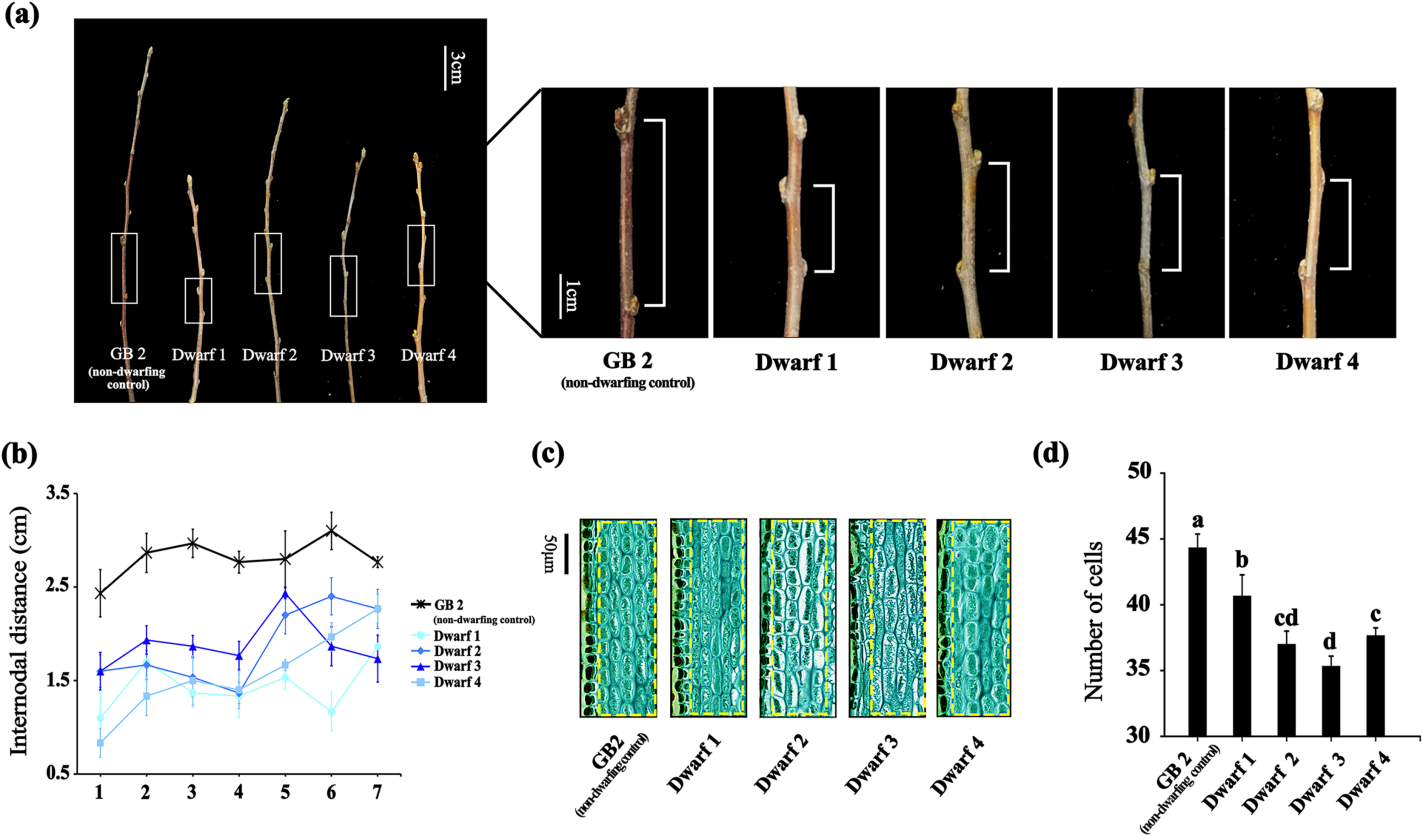
The phenotypes of the four dwarfing and control apple germplasms. (A) Shoot lengths of the four dwarfing and control apple germplasms. Bar, 3 cm. (B) Internodal distance of the four dwarfing and control apple germplasms. (C) Paraffin sections of shoot of the four dwarfing and control apple germplasms. (D) Statistical chart of cell number per unit area. Data are means ± SD of triplicate studies. Different lower-case letters indicate significant differences among germplasms according to Fisher’s LSD test (*p* < 0.05).

To investigate the underlying molecular mechanism of dwarfing in apple, we selected four dwarfing apple germplasms and a non-dwarfing apple germplasm used as control. Compared with GB2, the annual branches of the dwarfing apple germplasms exhibited significantly short nodular length (Fig. 1A). As the branch growing, differences of the nodular distance between the non-dwarfing and dwarfing apple germplasms became more apparent. The greatest differences were observed between nodes 3 and 5. At the third internode, GB2 had an internodal length of 2.97 cm, whereas that of dwarfing apple germplasms (Dwarf1-4 ) were 1.37, 1.53, 1.87 and 1.50 cm, respectively (Fig. 1B), indicating that dwarfing is caused by the shortened internodes.

To further determine whether the decrease in cell number or the inhibition of cell elongation is the cause of the dwarfing, we analyzed the longitudinal sections of stems with an optical microscope (Figs. 1C, 1D). Longitudinal sections of stems between the normal and dwarfing apple germplasms revealed clear difference in the cell number of cortical parenchyma cells. The cell number of dwarfing apple germplasms were significantly less than GB2. Therefore, shorter internodal lengths of dwarfing apple germplasms is the result of fewer cells.

### The annual branches of dwarfing apple germplasms have higher MT and MDA levels than that of GB2

**Figure 2:**
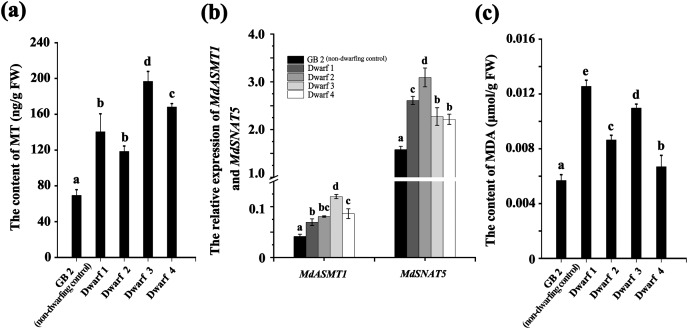
The annual branches of dwarfing apple germplasms have higher MT and MDA levels than that of GB2. (A) The melatonin level of the four dwarfing apple germplasms and control. (B) Relative expression level of *MdASMT1* and *MdSNAT5* in the four dwarfing and control apple germplasms. (C) The MDA content in the four dwarfing and control apple germplasms. Data are means ± SD of triplicate studies. Different lower-case letters indicate significant differences among germplasms according to Fisher’s LSD test (*p* < 0.05).

We measured the content of MT and MDA in GB2 and dwarfing germplasms. The MT contents in all the four dwarfing germplasms (Dwarf1-4) were significantly higher than that of non-dwarfing germplasm (Fig. 2A). Their contents were at least 1.7 times higher than that of the non-dwarfing germplasm, and the highest MT level could reach 196.827 ng·g^−1^, which is about three times higher than that of the GB2.

The relative expressions of *MdASMT1* and *MdSNAT5* were also significantly higher in the four dwarfing germplasms than that in the non-dwarfing germplasm. As shown in Fig. 2B, the relative expression of *MdASMT1* in four dwarfing germplasms were 1.66, 1.93, 2.87 and 2.07 times of higher that that in GB2, respectively. The relative expression of *MdSNAT5*, a rate-limiting enzyme for melatonin synthesis in mitochondria, showed a similar trend, and the level in four dwarfing germplasms were 1.64, 1.95, 1.43 and 1.40 times higher than that in GB2, respectively (Fig. 2B).

In addition, the content of MDA, indicating the degree of lipid oxidative damage, was also detected. Similarly, the MDA content in dwarfing germplasms were significantly higher than that of GB2 (0.00568 μmol·g^−1^ FW). Among the dwarfing germplasms, the MDA content in Dwarf1 (0.01255 μmol·g^−1^ FW) was the highest, which was 2.2 times higher than that of non-dwarfing germplasm, and the MDA content in Dwarf4 (0.00669 μmol·g^−1^ FW) was the lowest, which was 1.2 times higher than that of GB2 (Fig. 2C).

### The ABA level is higher in dwarfing apple germplasms than that of GB2

**Figure 3:**
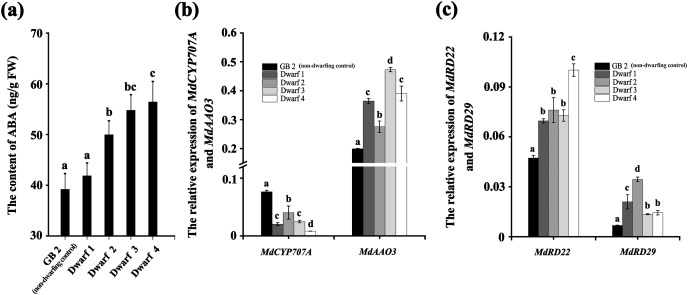
The ABA level is higher in dwarfing apple germplasms. (A) The ABA content in the four dwarfing and control apple germplasms. (B) Relative expression level of *MdCYP707A* and *MdAAO3* in the four dwarfing and control apple germplasms. (C) Relative expression level of *MdRD22* and *MdRD29* in the four dwarfing and control apple germplasms. Data are means ± SD of triplicate studies. Different lower-case letters indicate significant differences among germplasms according to Fisher’s LSD test (*p* < 0.05).

Likewise, the ABA content in all the four dwarfing apple germplasms were significantly higher than that in GB2. The ABA content in Dwarf2-4 were 49.949, 54.766, and 56.410 ng·g^−1^, respectively, while it was only 39.174 ng·g^−1^ in the GB2. Although the ABA content in Dwarf1 (41.848 ng·g^−1^) was lower than the other three dwarfing apple germplasms, it was still higher than that in GB2 (Fig. 3A). The expression of *MdRD22* and *MdRD29* (ABA inducible genes involved in ABA signaling) in four dwarfing apple germplasms were significantly higher than that in GB2 (Fig. 3C).

Furthermore, the relative expression of key genes involved in ABA biosynthesis and degradation were also analyzed in non-dwarfing and dwarfing apple germplasms. Compared with the GB2, the *MdAAO3* (a key gene in ABA biosynthesis) level in four dwarfing apple germplasms were significantly high, while the *MdCYP707A* (a key gene in ABA degradation) level was significantly lower than that of GB2 (Fig. 3B), which is another mechanism to lead to the enhanced ABA concentrations.

### The ZR level is lower in dwarfing apple germplasms than that in GB2

**Figure 4:**
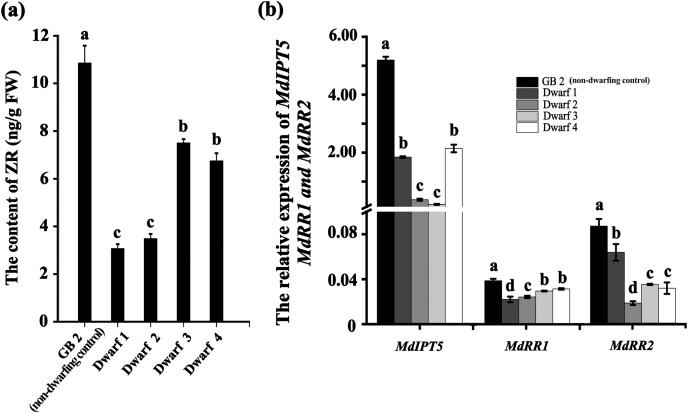
The ZR level is lower in dwarfing apple germplasms. (A) The ZR content of the four dwarfing and control apple germplasms. (B) Relative expression level of *MdIPT5, MdRR1* and *MdRR2* in the four dwarfing and control apple germplasms. Data are means ± SD of triplicate studies. Different lower-case letters indicate significant differences among germplasms according to Fisher’s LSD test (*p* < 0.05).

Plant height is primarily regulated by hormones, including GA, IAA, ZR and BR. We measured the contents of GA, IAA, ZR and BR in dwarfing and GB2 germplasms. Between them, the concentrations of IAA, GA3 and BR had no significan differences (Table S1). By contrast, the concentration of ZR was significantly lower in dwarfing germplasms than that of GB2. The ZR concentrations in the dwarfing apple germplasms (Dwarf1-4) were 3.071, 3.486, 7.504 and 6.750 ng·g^−1^, respectively which were only 28.3%, 32.1%, 69.1% and 62.2% of level of GB2 (10.857 ng·g^−1^) (Fig. 4A). The expression of *MdRR1* and *MdRR2* (ZR up-regulated genes) in four dwarfing apple germplasms were significantly lower than that in GB2 (Fig. 4B). We analyzed the expression of *MdIPT5* (a key gene in ZR biosynthesis), the relative expression of *MdIPT5* in the four dwarfing germplasms were significantly lower than that in GB2, and *MdIPT5* expression level in Dwarf3 was the lowest (Fig. 4B).

## Discussion

**Figure 5:**
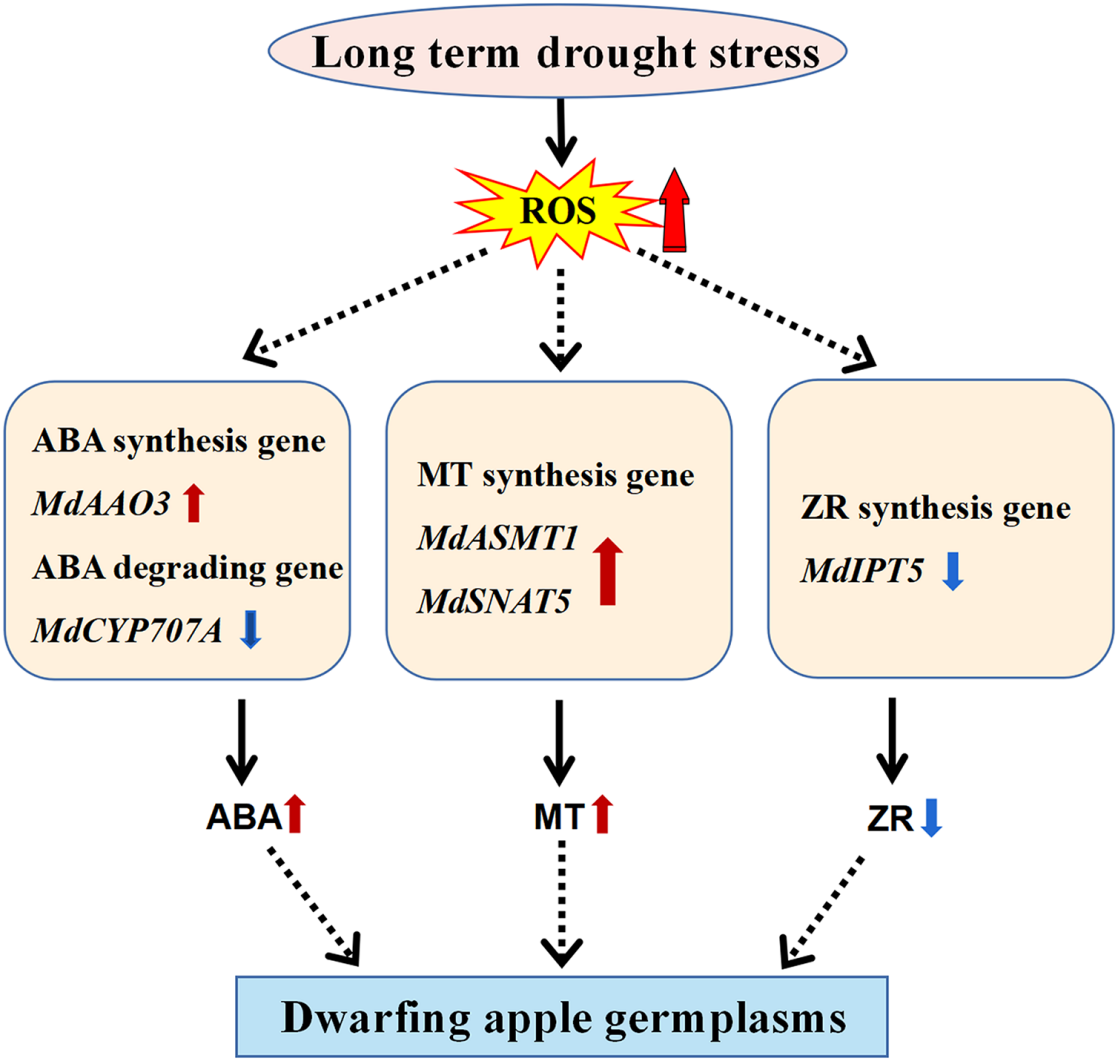
Model of the molecular mechanism that mediates dwarfing of apple germplasms. Enhanced content of melatonin, and ABA and reduced content of ZR induced by long-term drought stress resulted in the apple dwarfing phenotype. The red solid arrow indicates an increase and the blue solid arrow indicates a decrease. The black solid arrows denote direct activation.

Dwarfing is the major breeding trait targeted by researchers to increase apple yield and improve mechanical strengthening of the apple tree in the modern orchard (Bulley et al., 2005; Smolka et al., 2010; Yang et al., 2012). Due to the complex of the dwarfing process involved in the perennial woody apple tree, extensive studies have been carried out by researchers at the temptation to uncover its underlining mechanisms. For this purpose, we have collected four dwarfing apple germplasms in the semi-arid area of Xinjiang, China to explore the mechanisms of dwarfing process. Unexpected, the levels of all well-known phytohormones involved in plant dwarfing including IAA, GA3, and BR (Zheng et al., 2017; Zheng et al., 2018) did not show any correlation with the phenotypes of the four dwarfing apple germplasms. In contrast, the levels of stress-responsive molecules, MT and ABA, significantly increased in all of the dwarfing apple germplasms (Figs. 2A, 3A), inferring the dwarfing phenotype may be an evolutionary adaptation responding to the drought stress (Fig. 5).

The elevated level of MDA in the dwarfing apple germplasms indicates the significantly increased oxidative damage, possibly induced by long-term drought stress in semi-arid area of Xinjiang (Fig. 2C). This oxidative damage caused by the over-produced ROS, which in turn, triggers the synthesis of ROS scavenger, MT (Zheng et al., 2017) and the enhanced MT level has been detected in all dwarfing apple germplasms. The MT level actually is determined by both its synthesis and consumption. The expression of MT synthetic enzyme genes *MdASMT1* and *MdSNAT5* are significantly upregulated in all the dwarfing apple germplasms (Fig. 2B). The expression pattern of these genes correlate well with the MT level, confirming that MT synthetic genes contribute great to the MT content in the dwarfing apple germplasms. There are some inconsistent reports related to the role of MT in plant dwarfing. For example, the reduced melatonin leads to dwarfing transgenic rice (Hwang & Back, 2018) while the enhanced melatonin level has detected in transgenic tomato dwarfing lines (Wang et al., 2014). These inconsistences suggest the different roles of melatonin between monocotyledon and dicotyledon plants. In the perennial woody apple tree, high melatonin level is first repoted to associated with its dwarfing phynotype. As a ROS scavenger, melatonin level is highly regulated by environmental alterations and therefore it has a high chance to manipulate the dwarfing process associated with environmental changes.

The increased ABA level in the dwarfing apple germplasms may also result from the long-seasonal drought stress (Yoshida et al., 2021). The expression of *MdAAO3* (ABA synthetase gene), *MdRD22* and *MdRD29* (ABA signaling genes) are significantly upregulated while ABA degradation gene *MdCYP707A*, is downregulated in all of the dwarfing apple germplasms. These suggest that the increased ABA level is attributed to both its enhanced synthesis and reduced degradation (Fig. 3B).

Role of the increased ABA in apple dwarfing germplasms can be logically explained, *i.e*., the long-term drought stress induces ABA accumulation and therefore promotes stomatal closure. This leds to decrease photosynthesis by promoting ABA-mediated chlorophyll degradation (Asad et al., 2019) and reducing carbon dioxide in-take and therefore retards apple tree growth. Similarly, the pear trees which accumulated with ABA also show dwarfing phenotype after knockout of *PbPAT14* (Pang et al., 2019). The similar phenomenon of dwarfing phenotype due to ABA accumulation was also found in Gerbera hybrida and *Arabidopsis thaliana* (Yang, Worley & Udvardi, 2015; Ren et al., 2018). On the contrary, there are also reports on the positive role of ABA in regulating apple tree growth, that is, the reduced ABA and BR synthesis are found in transgenic dwarfing apple trees which overexpress *MdNAC1* (Jia et al., 2018). But it is not clear whether this dwarfing phenotype is mainly caused by the reduced ABA or BR. Li et al. (2015), have reported that the exogenous melatonin pre-application reduces drought-inducible ABA biosynthesis. It is likely that the exogenous melatonin decreases ROS burst, thus weakens stress signal and ABA biosynthesis. In our research, the long-term environmental drought stress induced both ABA and melatonin biosynthesis. Apple plants may adapt the stress for survival at the cost of retarded growth. The environmental stress can be converted into hormone signals including both ABA and melatonin, which triggered the ZT-regulated growth retard.

Kamboj et al. (1999) have observed that ZR, a zeatin precursor, is found to negatively regulate apple dwarfing. Our results showed that ZR content of all the apple dwarfing germplasms were significantly lower than control plant (Fig. 4A). The ZR content correlates well with the expression of its synthetase gene *MdIPT5* and the signaling genes *MdRR1* and *MdRR2*, which were lower in all of the apple dwarfing germplasms than control plant (Fig. 4B). This suggests that ZR biosynthesis is probably also involved with the apple dwarfing. Moreover, it is well documented that growth and development of plant are mainly influenced by CK signaling pathways (Kieber & Schaller, 2018; Mao et al., 2019; Li et al., 2021). In Arabidopsis, overexpression of *RR1* promotes stem growth (Hwang & Sheen, 2001). In the current study, the result indicates that high level of *MdIPT5* expression in non-dwarfing germplasm increases zeatin level to promote cell division and internode elongation, while low level of *MdIPT5* expression in the dwarfing germplasms results in defective zeatin and further reduced internode length. To reduce the ZR content might be an important strategy for apple to survive long-term drought stress *via* decreasing cell division and energy cost, retarding growth and promoting dwarfing (Ma et al., 2020; Zong et al., 2021).

Whether this stress-induced dwarfing phenotype can pass to the next generation or maintain in the propagated apple tree is currently unknown. However, many environment-inducted methylations for altered gene expression, selected in the evolution, is inheritable (Marfil et al., 2006; Keyte et al., 2006). This stress-tolerance dwarfing mechanism may also be a heritable strategy. But further research is needed to draw a conclusion.

## Conclusions

In summary, we have identified a potential molecular mechanism of apple dwarfing to adapt the drought in the semi-arid areas of Xinjiang, China. This mechanism is involved in not only the enhanced levels of melatonin and ABA, but also reduced content of ZR in different apple dwarfing germplasms. These hormone alterations are primarily caused by the long-lasting drought season which the apple dwarfing plants face to in Xinjiang, China. This is the first report to show a drought-associated dwarfing mechanism in apple trees. This dwarfing process is involved in altered gene expressions of phytohormones and antioxidant (ROS scavenger). These observations provide novel dwarfing mechanisms for apple breeding and production. In addition, this drought stress induced dwarfing phenotype in apple tree may be inheritable and to indetify this is the goal of our future study.

## Supplemental Information

10.7717/peerj.13008/supp-1 Supplemental Information 1 IAA,GA3 and BR content of GB2, Dwarf 1, Dwarf 2, Dwarf 3 and Dwarf 4.

10.7717/peerj.13008/supp-2 Supplemental Information 2 Primers used for qPCR. Primer sequences were designed by Primer 5 software according to the coding sequences from GDR (https://www.rosaceae.org/species/malus/all).

10.7717/peerj.13008/supp-3 Supplemental Information 3 qPCR detection of melatonin synthase gene expression in 5 germplasms.

10.7717/peerj.13008/supp-4 Supplemental Information 4 qPCR detection of gene expression of ZR and ABA metabolic pathways in 5 germplasms.

10.7717/peerj.13008/supp-5 Supplemental Information 5 qPCR detection of gene expression of ABA- and CK-regulated in 5 germplasms.

## Additional Information and Declarations

### Data Availability

The following information was supplied regarding data availability: The raw measurements are available in the Supplemental Files.
