# Supplementary material for: Potential roles of melatonin and ABA on apple dwarfing in semi-arid area of Xinjiang China
Source: PeerJ. 2022 Mar 31;10:e13008. doi: 10.7717/peerj.13008 (PMC8977067; doi:10.7717/peerj.13008)
Supplement: Supplemental Information 1 [file peerj-10-13008-s001.docx]

| **Table S1.** IAA,GA3 and BR content of GB2, Dwarf 1, Dwarf 2, Dwarf 3 and Dwarf 4 |
| --- |
| \| **Sample name** \| **Hormones content ( ng .g^-1^FW)** \| \| \| \| --- \| --- \| --- \| --- \| \| **IAA** \| **GA3** \| **BR** \| \| GB2 \| 31.96321 \| 5.49446 \| 5.12236 \| \| 37.55081 \| 5.03699 \| 5.39857 \| \| 36.61207 \| 5.96748 \| 4.68591 \| \| Dwarf 1 \| 49.49553 \| 5.37634 \| 4.04879 \| \| 50.29941 \| 5.59082 \| 4.53848 \| \| 41.36192 \| 5.18133 \| 4.64330 \| \| Dwarf 2 \| 10.18290 \| 5.68887 \| 4.67522 \| \| 11.47752 \| 5.04795 \| 4.02115 \| \| 11.93550 \| 5.91583 \| 4.32598 \| \| Dwarf 3 \| 36.52790 \| 7.28827 \| 4.55925 \| \| 33.08596 \| 8.17801 \| 4.72891 \| \| 38.86983 \| 8.52277 \| 5.11068 \| \| Dwarf 4 \| 22.42430 \| 9.19635 \| 6.40702 \| \| 24.81415 \| 8.63463 \| 6.26238 \| \| 20.40502 \| 8.33955 \| 5.54855 \| |
