# Supplementary material for: Potential roles of melatonin and ABA on apple dwarfing in semi-arid area of Xinjiang China
Source: PeerJ. 2022 Mar 31;10:e13008. doi: 10.7717/peerj.13008 (PMC8977067; doi:10.7717/peerj.13008)
Supplement: Supplemental Information 2 — Primer sequences were designed by Primer 5 software according to the coding sequences from GDR (https://www.rosaceae.org/species/malus/all). [file peerj-10-13008-s002.docx]

| **Table S2.** Primers used for qPCR |
| --- |
| \| **Gene name** \| **Accession**  **number** \| **Forward primer** \| **Reverse primer** \| \| --- \| --- \| --- \| --- \| \| *MdASMT1* \| MD05G1308800 \| CTGTTTTCCTCAAGCTGACT TTG \| CTCTTCCAGTGACCACAACCATC \| \| *MdSNAT5* \| [MD09G1249800](http://www.rosaceae.org/feature/MD09G1249800) \| TGGGCGACGATAGTGGAAG AAATAA \| TCCGACAAGTATCCTTCACTGCGA \| \| *MdAAO3* \| MD07G1238600 \| GGATCCATGGCGGCAGCTACAGGGGT \| GTCGACCTAGCTGGCATCAAAGTCTA \| \| *MdCYP707A* \| MD01G1051900 \| ATCTATTGCTGGGTGTCCTATGTGG \| CGATAACCTATACGGTACTGCGGTA \| \| *MdIPT5* \| MD16G1183400 \| TTACACTGTGAAGCGAGTGGTAG \| GTCGGAAGAATGGAGGGAATT \| \| *MdRD22* \| MD15G1098800 \| TGATGGGGTGAATGTTAAAG \| GAACCCAGACAACATGATCA \| \| *MdRD29* \| MD07G1268800 \| CCAAATTACCATGCCTCAAC \| CCTTGGACTTGTACTCTCCC \| \| *MdRR2* \| MD16G1159400 \| ATGATATGTTTAACGAGTTACAG \| GCCTTCGTCCATTGAGAACAGAG \| \| *MdRR1* \| MD13G1159700 \| ACACTCCATACACGGTAGCCT \| GGATTCCTGCTTTGTCCAC \| |

**Note:** Primers sequences were designed by Primer 5 software according to the coding sequences from GDR (https://www.rosaceae.org/species/malus/all).
